# Anomalous electrical conduction and negative temperature coefficient of resistance in nanostructured gold resistive switching films

**DOI:** 10.1038/s41598-020-76632-y

**Published:** 2020-11-12

**Authors:** M. Mirigliano, S. Radice, A. Falqui, A. Casu, F. Cavaliere, P. Milani

**Affiliations:** 1 CIMAINA and Department of Physics, Università Degli Studi Di Milano, via Celoria 16, 20133 Milano, Italy; 2 NABLA Lab, Biological and Environmental Sciences and Engineering (BESE) Division, King Abdullah University of Science and Technology (KAUST), Thuwal, 23955-6900 Saudi Arabia

**Keywords:** Condensed-matter physics, Materials for devices, Nanoscale materials

## Abstract

We report the observation of non-metallic electrical conduction, resistive switching, and a negative temperature coefficient of resistance in nanostructured gold films above the electrical percolation and in strong-coupling regime, from room down to cryogenic temperatures (24 K). Nanostructured continuous gold films are assembled by supersonic cluster beam deposition of Au aggregates formed in the gas phase. The structure of the cluster-assembled films is characterized by an extremely high density of randomly oriented crystalline nanodomains, separated by grain boundaries and with a large number of lattice defects. Our data indicates that space charge limited conduction and Coulomb blockade are at the origin of the anomalous electrical behavior. The high density of extended defects and grain boundaries causes the localization of conduction electrons over the entire investigated temperature range.

## Introduction

Granular metallic films (GMFs) consist of random networks of metal clusters or nanoparticles, with different size and structure, separated by a dielectric matrix (either vacuum or a non-conducting material)^1–3^. The electrical properties of GMFs are strongly dependent on the coupling between adjacent metallic units and the transition from non-metallic transport to metallic conduction has been actively studied by varying their density from very diluted (weak-coupling regime) to particle structural percolation (strong-coupling regime)^3–9^. Systems in weak-coupling regime have received particular attention in order to understand the role of defects and discontinuities in determining the non-metallic behavior, whereas systems in strong-coupling regime are reported to be ohmic with conventional transport mechanisms typical of polycrystalline metallic films^10–13^.

Random networks of metallic nanowires/nanoparticles in a polymeric matrix or passivated by shell of ligands or oxide layers have gained a renewed interest for the fabrication of non-linear circuital elements such as memristors and resistive switching devices for analog computing and neuromorphic data processing^14–18^. These systems are in the weak-coupling regime and their electrical behavior is determined by the formation/destruction of conducting junctions between isolated nanoparticles conferring neuromorphic properties to the networks^14–17,19–21^.

Recently we showed that granular systems in strong-coupling regime consisting of continuous cluster-assembled gold films produced by the assembling of unprotected clusters, also show resistive switching^22,23^. Their structure is characterized by the random stacking of differently shaped crystalline clusters directly connected by junctions of different cross sections with an extremely high number of defects and grain boundaries^22,23^.

Here we report that continuous cluster-assembled gold films, although in strong-coupling regime, show non-metallic electrical conduction and negative Temperature Coefficient of Resistance (TCR) within 24–300 K temperature range. The observed behavior indicates that conduction mechanisms typical of insulators or highly disordered semiconductors are occurring. Remarkably, the resistive switching activity of these systems is maintained down to cryogenic temperatures.

## Experimental methods and characterization

**Figure 1:**
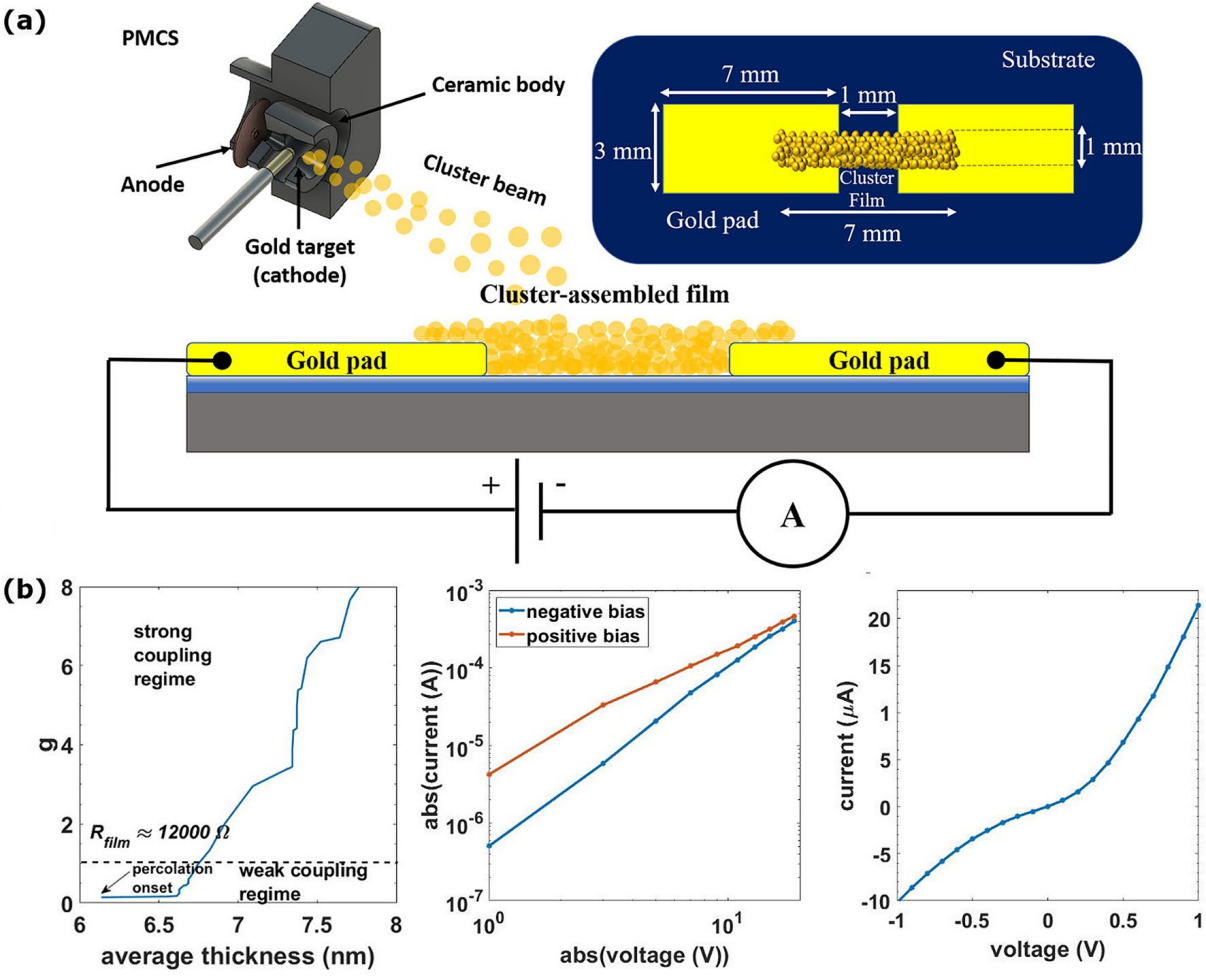
(**a**) Schematic view (not to scale) of a two-terminal device constituted by thermally deposited gold electrodes bridged by a cluster-assembled gold film. The blue region beneath the gold films is a silicon oxide layer. Electrical characterization is performed with an ammeter connected in series to a voltage source at room temperature. On the top left, a scheme (not to scale) of the cluster source (PMCS) used to generate the supersonic cluster beams. On the top right, a scheme with the device dimensions. The gold pads have a rectangular shape 7 mm × 3 mm, spaced by 1 mm and bridged by a 1 mm × 7 mm cluster film. The gold pads are 100 nm thick. (**b**) Left panel: evolution of the dimensionless tunnel conductance g beyond the percolation threshold, as function of the thickness of the film. Central panel: I–V curves in double logarithmic scale measured at RT. Red curve under positive bias, the blue one under negative bias. Right panel: I–V curve for voltage in the range − 1 V to 1 V.

Nanostructured Au films were produced by a supersonic cluster beam deposition apparatus equipped with a Pulsed Microplasma Cluster Source (PMCS)^24^, as described in detail in reference^23^. Figure 1a shows a schematic view of the Au cluster formation and deposition process. Au clusters with a bimodal log-normal mass distribution peaked around 5 nm are formed in an Argon atmosphere after the plasma ablation of a gold target^23,25^,the cluster-Argon mixture is then expanded into the vacuum to form a supersonic beam directed on a silicon substrate with a thermally grown oxide layer^22,23^. Clusters are deposited between two gold electrodes previously fabricated by thermal evaporation (Fig. 1a). Supersonic cluster beams are characterized by high collimation: this guarantees the patterning of films with a high lateral resolution by using stencil masks, as described in^23,26^. The amount of deposited material is measured by a pre-calibrated quartz microbalance, the evolution of the electrical resistance of the films is monitored in situ and ex situ in a two-probe configuration.

In situ electrical characterization in a range from room temperature (RT, 300 K) down to 24 K has been performed in vacuum (10^–5^ mbar) on films mounted on a copper cold finger of a helium mechanical cryocooler. The film overall features have been investigated by high resolution TEM (HRTEM), using a spherical aberration-corrected microscope with an ultimate point resolution of 0.07 nm^27^.

We characterized continuous films with an average thickness ranging from 15 to 30 nm, and resistance, before switching activation^21–23^, varying from 80 Ω to 1000 Ω. This range belongs to strong-coupling regime where ohmic behavior and positive TCR are usually observed in granular films^3,11,28^. The value of the dimensionless tunnel conductance ${\text{g}} = {\text{ h}}/2{\text{e}}^{2} {\text{R}}_{{\text{t}}} \left( {{\text{T}} \to \infty } \right)$, where ${\text{R}}_{{\text{t}}} \left( {{\text{T}} \to \infty } \right)$ is the average tunnel resistance of the granular system at high temperature, discriminates the weak-coupling regime (${\text{g}} < 1$) from the strong-coupling one (${\text{g}} > 1$)^3,6^. In Fig. 1b (left panel) we show the evolution of g, obtained by approximating ${\text{R}}_{{\text{t}}}$ to the resistance at room temperature, after the onset of percolation threshold, as function of the cluster-assembled film thickness^23^. The fast increase of g is due to the high rate of deposition and the formation of connections among the clusters that open new conductive paths. Figure 1b shows the transition to the strong-coupling regime at a thickness value of roughly 7 nm.

Figure 1b (central panel) shows the room temperature I–V characteristics of the cluster-assembled film under positive and negative bias voltage in double logarithmic scale. A clear departure from an ohmic behavior is evident, with an asymmetry under positive and negative bias (red and blue curve respectively). The slope change observed in the curves is caused by the presence of switching events during the application of the voltage ramp^22,23^. The coexistence of non-ohmic behavior with switching events in Au cluster assembled-films has been reported and discussed in^22^. Figure 1b (right panel) shows the room temperature I–V curve in linear scale for small applied voltages (values in the range − 1 V to 1 V). Also in this case, the trend deviates form that expected for an ohmic conduction.

**Figure 2:**
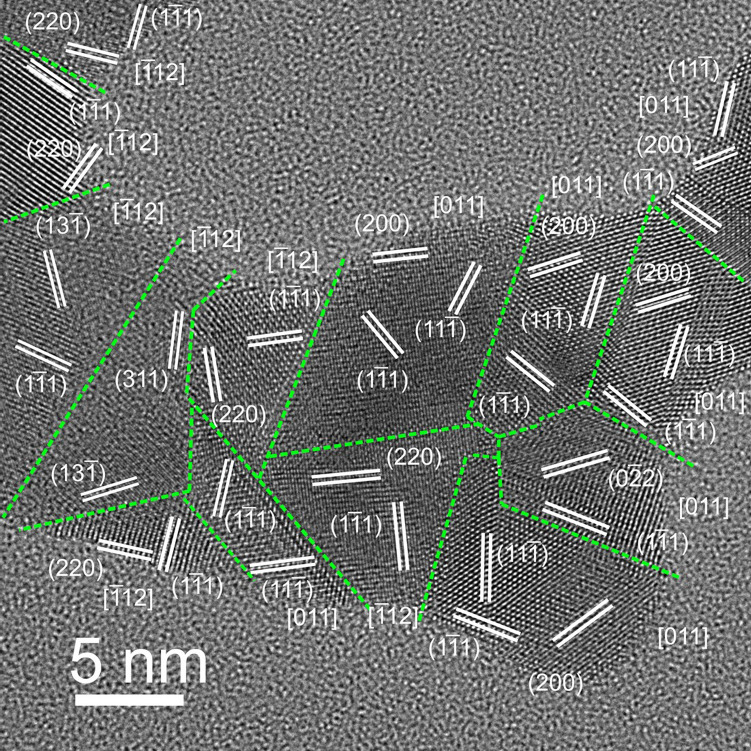
HRTEM image of a typical region of the film, in sub-monolayer regime. Different crystal domains constituting the film are separated by grain boundaries. For each single crystal domain, characteristic interplanar distances (hkl) and the corresponding zone axis [hkl] are displayed, after determination by local 2D-Fourier analysis of the relevant region of interest in the HRTEM image. The films clearly show an overall structure constituted by gold crystal domains, concomitantly allowing to rule out the presence of ordered phases other than metallic gold.

HRTEM analysis highlights the typical features of the film from a structural point of view (Fig. 2), namely its nanocrystalline, disordered nature. We characterized a film closed to percolation threshold, structural analysis performed by 2-Dimensional Fast Fourier Transform (2D-FFT) indicates that multiple, distinct crystal domains are formed, and their boundaries within the film are outlined by dashed lines. The size and shape of the domains are highly irregular and characterized by the presence of defects and with the smaller domains being less than 5 nm wide.

## Results and discussion

It is usually assumed that the electrical conduction properties of granular films in the strong-coupling regime are analogous to those of polycrystalline metallic films^6,29^. The observed departure from an ohmic behavior at room temperature in continuous films resulting from the stacking of naked highly defective gold nanocrystals is unexpected. Our data suggest that different conduction mechanisms are taking place, dictated by the extremely high density of randomly oriented crystalline nanodomains and grain boundaries of the films (Fig. 2).

In the case of two-terminal devices based on semiconductor or insulating layers, information on the microscopic mechanisms determining the current–voltage characteristics can be extracted by considering the parameter $\gamma = \frac{{{\text{dln}}\left( {\text{I}} \right)}}{{{\text{dln}}\left( {\text{V}} \right)}}$, where ${\text{ln}}\left( {\text{I}} \right)$ and ${\text{ln}}\left( {\text{V}} \right)$ are the logarithms of the current and of the applied voltage, respectively^30,31^. The analysis is carried out by plotting γ against ${\text{V}}^{\frac{1}{2}}$, since this curve has a well-defined trend for different mechanisms such as ohmic, space charge limited conduction (SCLC), Schottky, Poole–Frenkel, tunnelling, etc.^3,30^.

**Figure 3:**
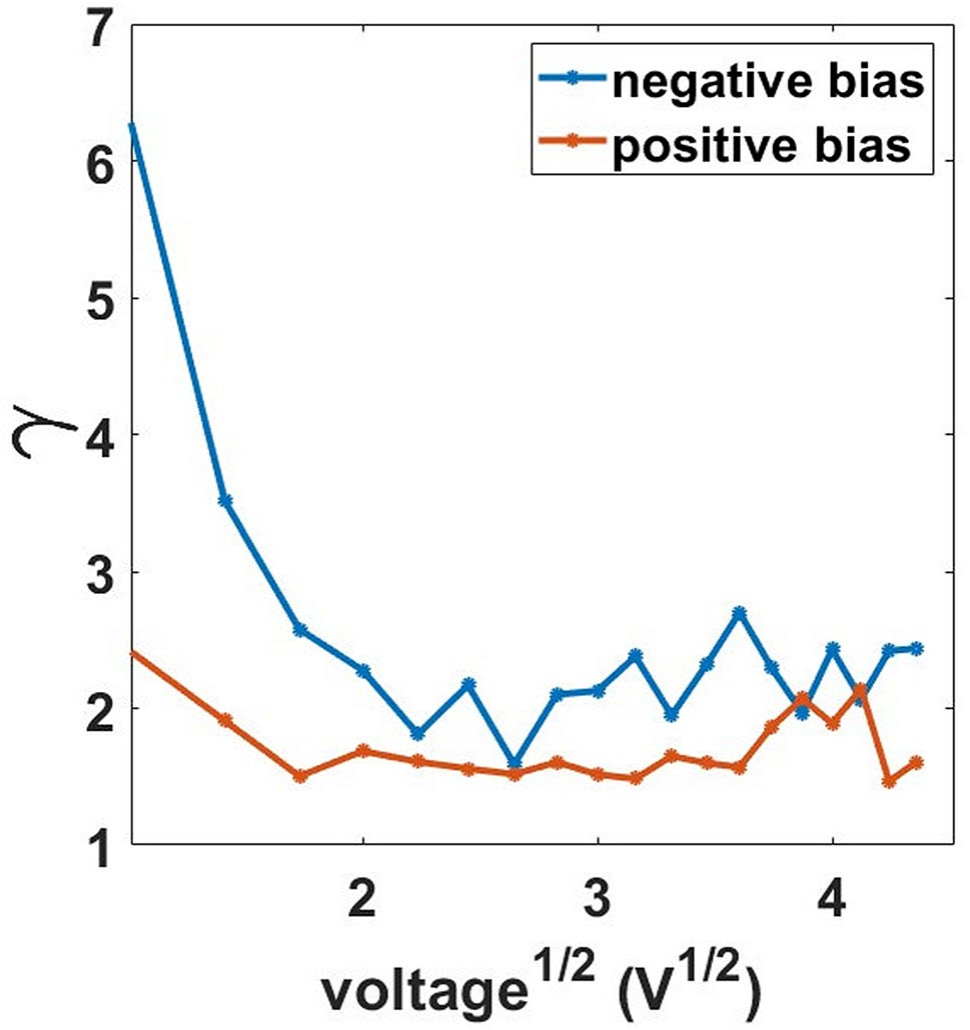
γ vs. the voltage square root for the increasing and the decreasing voltage branches of the I–V curve of a 15 nm thick cluster-assembled film.

The trend of the gamma parameter for our films is reported in Fig. 3 showing a transient (blue curve) before stabilizing around roughly 2, which is typical of SCLC^30,32,33^. In this regime, the free carrier density is low and the electrical conduction is usually determined by the charges injected from ohmic electrodes^30^.

In order to exclude the contribution of contact resistance, we tested atom-assembled thin films with similar thickness and geometry at RT. We found the standard ohmic behavior^23^. We can also exclude a contribution of the contact resistance at low temperatures since we did not observe a Schottky conduction contribution in the gamma curves, as one expects from contact resistance^34^.

In systems characterized by SCLC, ohmic conduction is usually observed at low bias voltages, due to the presence of a small fraction of thermally generated carriers^32^. Cluster-assembled gold films show a non-linear I–V curve even at very low voltages (Fig. 1b left panel) suggesting that different concurrent mechanisms contribute to determine a SCLC regime and to lower the free electron density. Coulomb blockade^9,35^ and defect localization effects^3,13,36^ could be possible causes for the low concentration of free carriers in our systems, due to their overall disordered crystal structure, which manifests as a very high density of grain boundaries as observed by HRTEM (Fig. 2).

To gain a deeper insight about the phenomena involved in the conduction process of cluster-assembled Au films and to discern among different mechanisms, we investigated the evolution of electrical conduction with temperature^9,37,38^.

**Figure 4:**
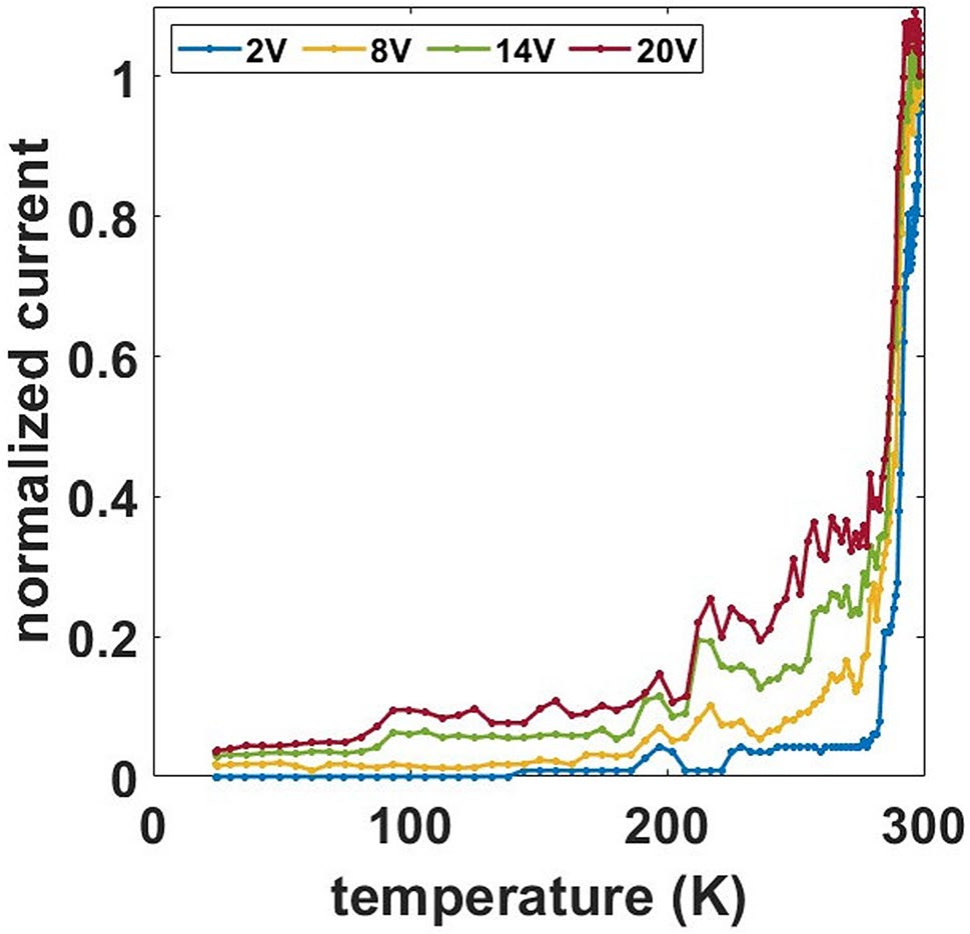
Current normalized to the value measured at RT as function of temperature for different applied voltages of a 25 nm thick sample. The sawtooth-like shape of curves at high voltages is due to switching events.

Figure 4 shows the temperature dependence of the current at different applied voltages normalized to the value measured at RT. We observe a steep decrease of the current in the range between RT and 250 K; from 250 to 24 K the decrease continues with a lower slope. The trend of the current for different applied voltages is qualitatively similar.

In metallic systems, finite electrical resistivity arises due to scattering processes from impurities or various thermal excitations^2,39–41^. The scattering events can be considered as statistically independent and thus additive, leading to the Matthiessen’s rule, where any thermally induced scattering simply increases the resistivity ρ(T)^42,43^. This corresponds to a positive Temperature Coefficient of Resistivity (TCR), i.e. dρ/dT > 0. Figure 4 clearly show that cluster-assembled gold films are characterized by a trend of resistance with temperature typical of non-metallic systems.

**Figure 5:**
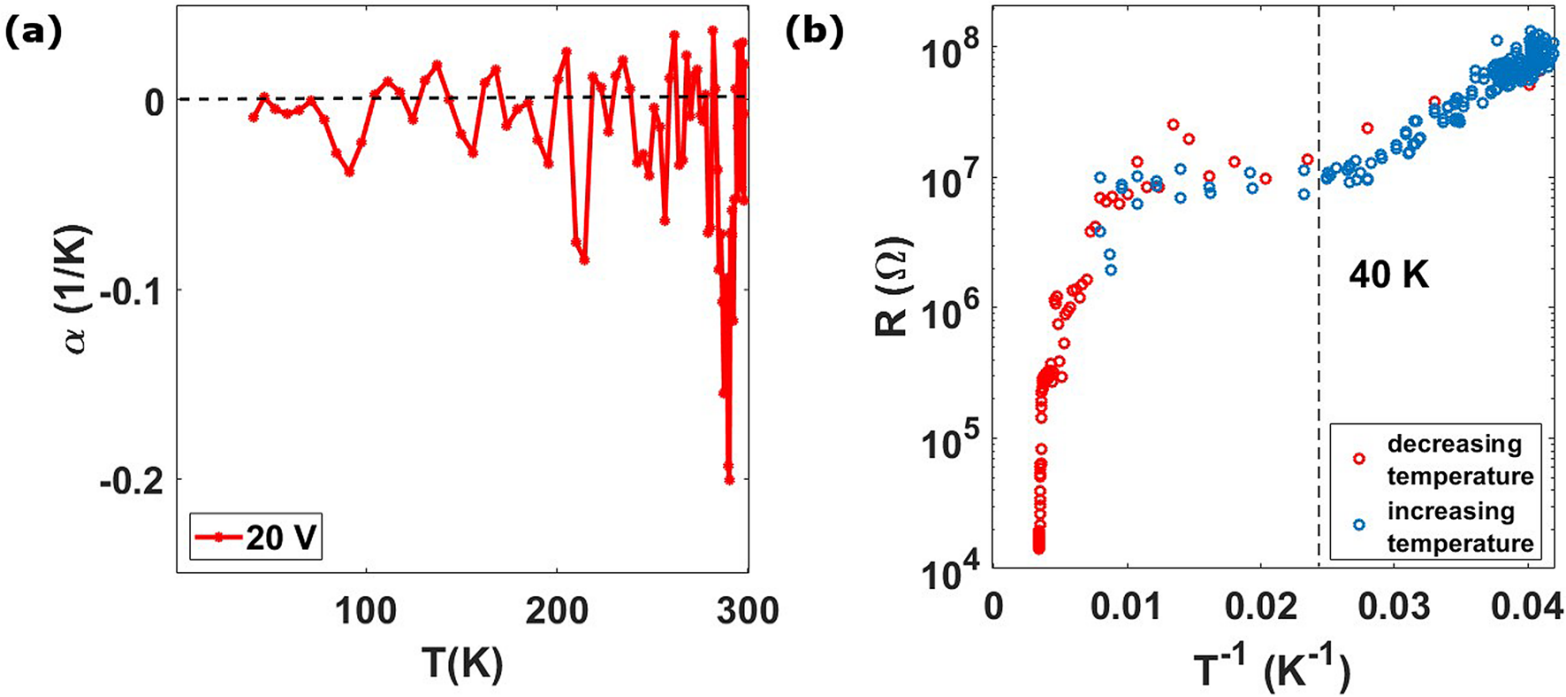
Data of a 25 nm thick cluster-assembled film. (**a**) the temperature coefficient of resistivity (TCR) for the curve measured upon the application of 20 V. (**b**) Resistance as a function of the inverse of the temperature, both for cooling (red circles and blue circles respectively) and heating (cross) cycles, in logarithmic scale. An Arrhenius-like trend is recognizable only for temperatures below 40 K.

Figure 5a shows that cluster-assembled gold films have a negative TCR, in particular near RT, the oscillation around zero at lower temperatures can be ascribed to the presence of switching events of amplitude smaller than the resistance temperature variations^22,23^.

These results are unexpected for a nanostructured metallic film in the strong-coupling regime: to the best of our knowledge only discontinuous gold ultrathin films and layers of molecularly linked gold nanoparticles have been reported to show non-metallic electrical conduction with temperature^5,6,12,28^, although not in such a large temperature range. Self-assembled films of CnS2-linked Au nanoparticles have electrical properties ranging from insulating to metallic-like depending on the separation of the Au building blocks^10^. In the insulating regime, electric transport occurs through cooperative electron tunneling (co-tunneling) at low temperatures, variable-range hopping (VRH) at intermediate temperatures, and Arrhenius-type behavior at high temperatures^36^. The weight of each of these contributions depends on both the interparticle separation and the spatial organization.

Similarly, discontinuous films composed by irregularly shaped gold islands, assembled by atom deposition, with density close to the percolation threshold^6,11^ show non-metallic transport strongly influenced by local disorder causing variations in the tunnel junction gaps and in the Coulomb blockade energies, due to island size fluctuations and offset charges^44^. A conduction percolation (co-percolation) model is applied to determine the total electrical current through the film as a function of both temperature and bias voltage^9^. The flowing of current is described as a percolation process through the ramified metallic islands. Unlike the case of hopping regime, in this case the high degree of disorder is related to the wide distribution, without mutual correlation, of the island electrostatic charging energy and of the parameters that characterize different tunnel junctions^6,9^. Increasing the island density till the reaching of strong-coupling regime, an ohmic electric transport is observed^6,9,28^.

Various types of disorder are considered at the origin of a negative TCR in ultrathin discontinuous films: (1) variations in the tunnel gaps between adjacent islands; (2) variations in the size and shape of the islands; (3) random offset induced by trapped impurity charges in the substrate^9,42^. In our case, cluster-assembled films are continuous and in the strong-coupling regime, however they do not show the electrical behavior typical of continuous metallic films. We suggest that this is due the extremely high concentration of defects and grain boundaries slicing the crystal domains that constitute the films: upon deposition on the substrates, gold clusters formed in the gas phase do not lose their individuality and give rise to the multidomain structure, as confirmed by HRTEM analysis^22^ ( see Fig. 2). This kind of spatially extended disorder is substantially different from what observed in polycrystalline metallic films grown by atom deposition, where the density of grain boundaries is much lower compared to that we find in our systems^45^.

Figure 5b displays the resistance vs the inverse of the temperature in logarithmic scale, showing that an Arrhenius-like behavior, characterized by a relation ${\text{R}}\left( {\text{T}} \right) \propto {\text{exp}}\left( {\frac{{{\text{T}}_{0} }}{{\text{T}}}} \right)$, is not detected except for temperatures below 40 K. The observed behavior deviates from a pure hopping conduction like that in the Efros-Shklovskii model^3,9,46^ for discontinuous films. On the other hand, the co-percolation model successfully describes the non-Arrhenius behavior of the electrical resistance at low bias voltage^6,9^, even if different mechanisms, such as Anderson localization, could contribute to the overall electrical behavior observed^36,47^. We also note that the trend is reversible, i.e. the resistance curve obtained during the cooling coincides well with that obtained during the heating, strongly suggesting that the observed behavior is not related to a phase transition but only to electronic properties.

**Figure 6:**
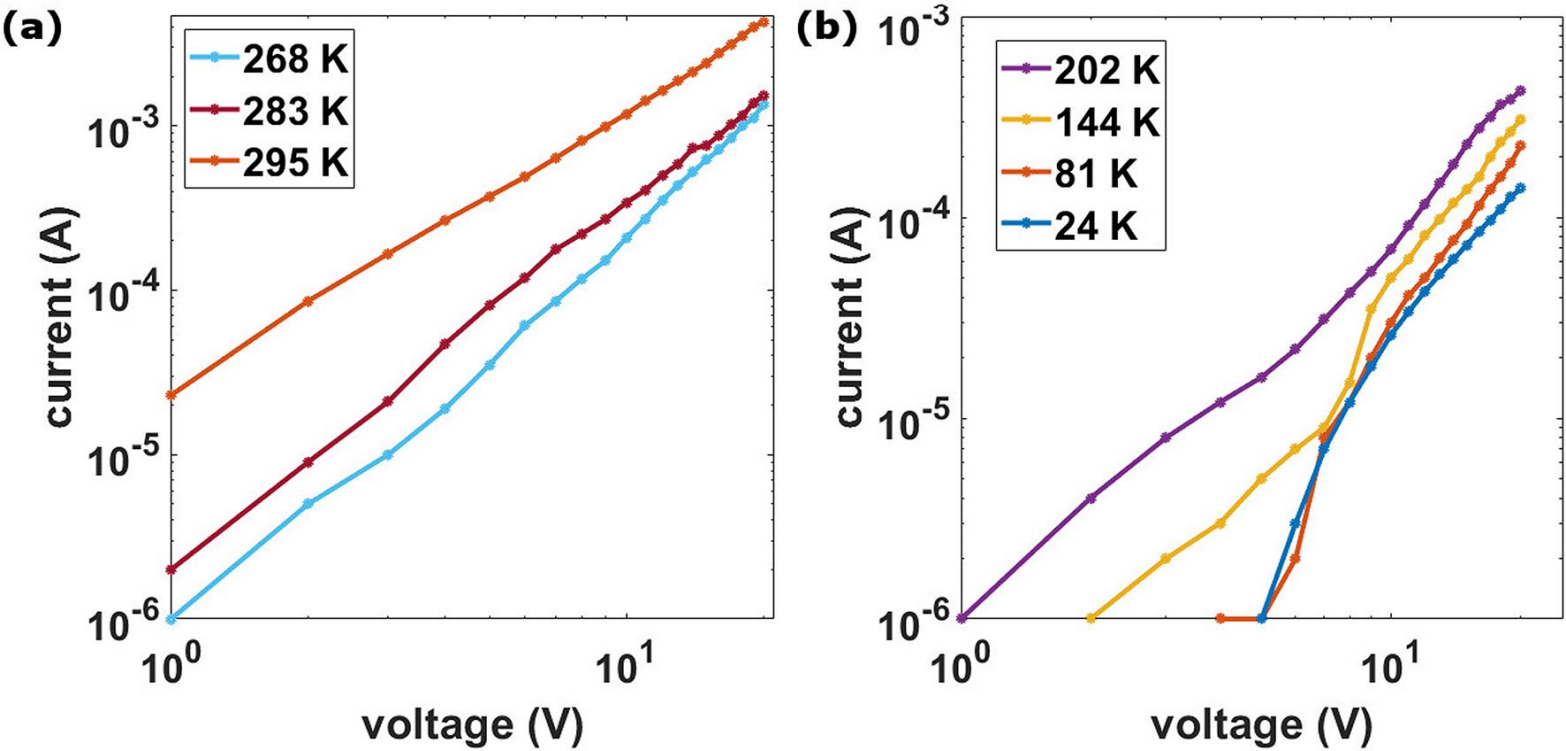
Data of a 25 nm thick cluster-assembled film. (**a**) I–V curves for different temperatures in the range 295 K to 268 K. (**b**) I–V curves in the range 202 K to 24 K.

The co-percolation model predicts not only the non-Arrhenius behavior but also a power law I–V characteristic (see Fig. 1b). Figure 6a shows the I–V characteristics in the temperature range 295 K to 268 K. The trend is constantly linear in double logarithmic scale and unaffected by temperature change except for a higher resistance measured at lower temperatures. On the other hand, in Fig. 6b the curves show steep slope variation for temperatures lower than 144 K. Although the trend slightly deviates from a pure power law, this agrees with the co-percolation model considering the occurrence of Coulomb blockade at cryogenic temperatures^9,35^.

**Figure 7:**
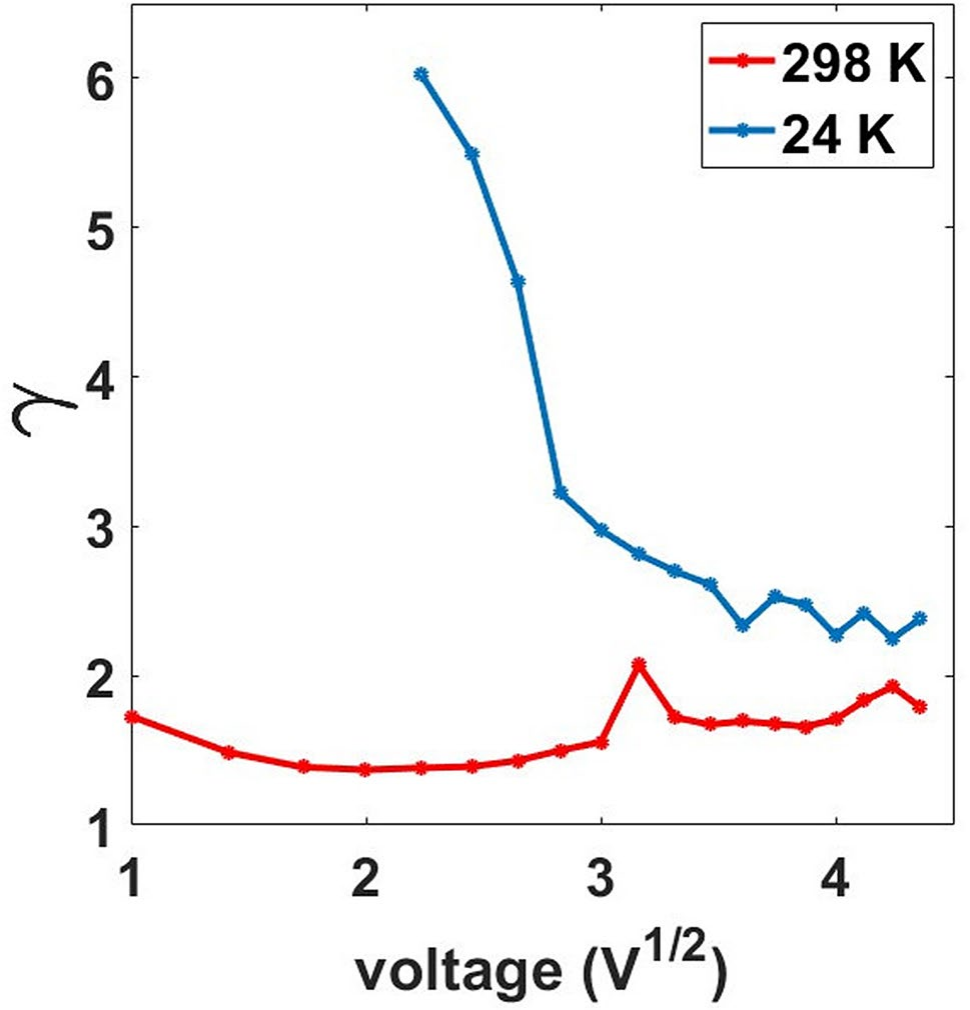
γ parameter as a function of the square root of the voltage of a 15 nm thick sample. The graph shows the evolution of γ for the positive branch in the I–V curve from 295 to 24 K. At low voltages (lower than 2 V) the current is suppressed; the curve shows the parameter values for voltage values greater than 2 V.

At low temperatures we also notice that the γ parameter explores values slightly larger than 2. Figure 7 shows the evolution of the γ parameter from 298 to 24 K. This agrees with the observation of higher resistance states at low temperatures and indicates that the variation of thermally generated carriers is at the origin of the effects observed at low voltages. In addition, we underline that the resulting parameter γ is not compatible with a pure SCLC, but it reflects the dependence of the current by an external voltage in a medium with low density of free carriers and electrostatic phenomena hampering charge motion^9^. This further indicates the high density of defects and the disordered configuration can alter the electronic properties in the metallic film, reducing the mobility for the conduction electrons. The presence of grain boundaries and defects modify the electronic band structure of a metal^48^ and its electrical conduction properties^42^. In our case the defect and grain boundaries densities are extremely high, compared to what observed in polycrystalline films^48^ thus causing substantial electronic localization, the formation of space charge and hence the SCLC trend.

In order to provide further elements to highlight the role of the high density of grain boundaries and defects in determining the conduction regime, we have characterized the behavior of films under constant bias in the time. Since cluster-assembled gold films exhibit resistive switching (RS) phenomena^21–23^, the evolution of the RS activity with temperature can provide useful elements.

**Figure 8:**
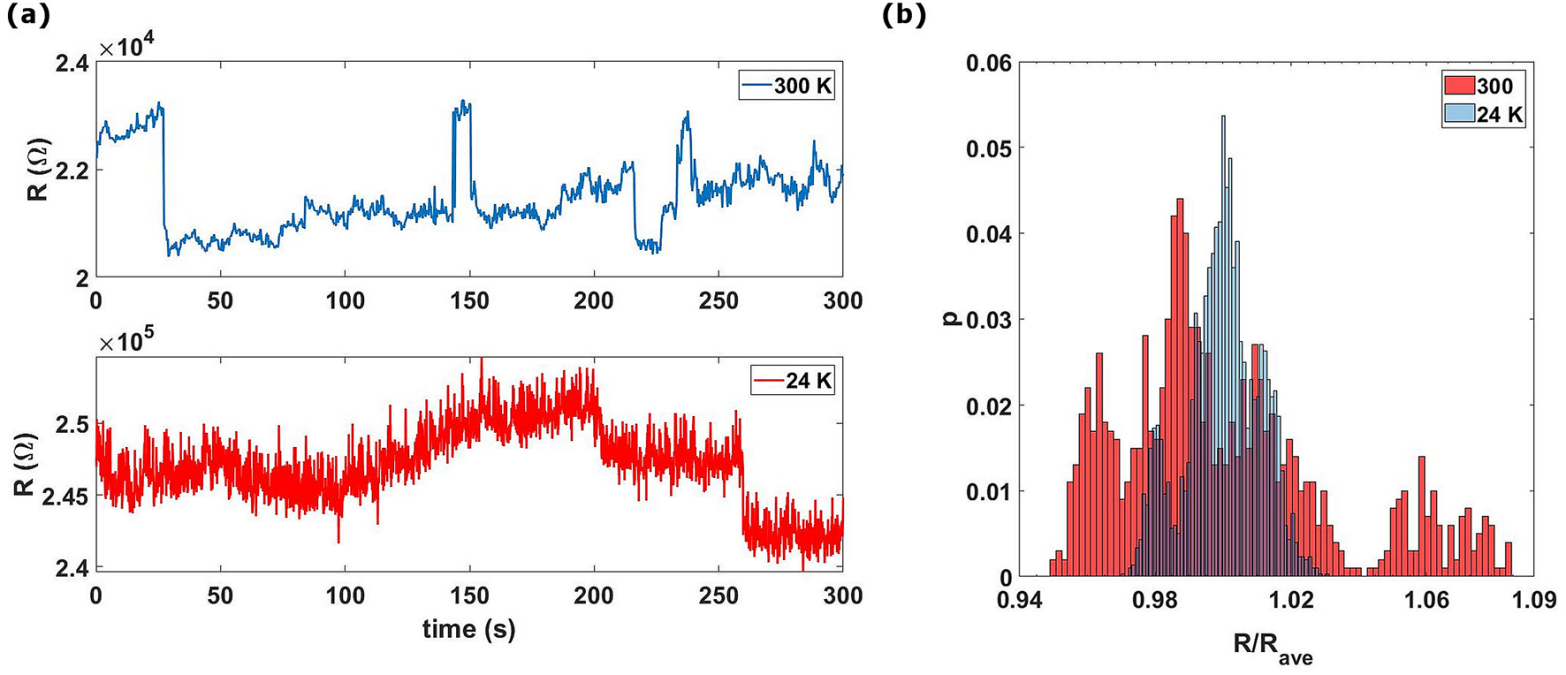
(**a**) The resistance-time graph, of a 15 nm thick sample, a 300 K (blue curve) and at 24 k (red one). (**b**) Distribution of the resistance values normalized by their average for the measurements carried out at constant voltage (5 V) at RT (red data) and at 24 K (blue data).

In Fig. 8a we show a typical resistance-time graph of the evolution of the resistance under 5 V bias. We add the histogram (Fig. 8b) of the resistance, measured at both RT and 24 K upon the application of a constant voltage, for a duration of 300 s. The different peaks in the histogram are due to the different resistance levels explored during the resistive switching phenomena. Remarkably we observe a substantial RS activity at 24 K spanning a lower number of levels compared to that at RT. We can interpret these results by considering that the flow of electric current causes the rearrangement of domains grown randomly with their related lattice defectivity and grain boundaries. As a consequence, concomitant dynamical creation and destruction of pathways with variable resistance occur through the rearrangement of defects^16,20^. This process is favored at RT by the high mobility of atoms and atomic planes^49^, while the latter is reduced at cryogenic temperatures.

The persistence of RS events at cryogenic temperatures is unexpected and it could be related to the structure of cluster-assembled films characterized by a landscape crowded of defects and interconnects between grains resulting in an assembly of interacting nanojunctions^44,48,50^. Multiple conductance states are observed in single metallic nanojunctions at cryogenic temperatures^51,52^ with electrical conductions characterized by discrete steps of conductance involving Coulomb blockade phenomena both in increasing and decreasing resistance^51,53^. Cluster-assembled gold films can be then considered as an assembly of nanojunctions connected in series and in parallel, thus displaying a collective electrical behavior resulting in resistive switching phenomena^20,22,54^. This confirms the facts that high densities of grain boundaries and nanojunctions can significantly alter the electronic properties, favoring the onset of non-ohmic conduction mechanisms. Atomic rearrangement present under the application of high voltage bias contributes to the rearrangement of grain boundaries responsible for the switching events. The observed mechanism is substantially different from what observed in random networks of nanowires where ionic transport is involved^14,15,17,18,55^. In our case the highly correlated re-arrangement of grain boundaries changes the local conductivity as observed in single metallic nanowires^56^.

## Conclusions

In summary, cluster-assembled continuous Au films in strong-coupling regime exhibit a non-metallic electrical conduction and a negative TCR over a range from RT to cryogenic temperatures. Our data can be explained by considering the coexistence SCLC and Coulomb blockade phenomena, similarly to what observed in highly disordered semiconductor or insulator films. Of primary importance for the understanding of the microscopic mechanisms responsible for these puzzling electrical properties is the influence of an extremely high density of grain boundaries and lattice defectivity on conduction electron localization.

Our results highlight that cluster-assembled gold films are a challenging platform for exploring the fundamental role of extended nanoscale defects on electron localization and transport mechanisms, and for the fabrication of resistive switching devices that can operate over a wide temperature range with interesting non-linear electrical properties which could be exploited for neuromorphic data processing^22,57,58^.
